# Structural correlates of aphasia severity, cognitive impairment, and outcome after stroke

**DOI:** 10.1016/j.nicl.2026.103954

**Published:** 2026-01-19

**Authors:** Célise Haldin, Hélène Lœvenbruck, Céline Piscicelli, Valérie Marcon, Shenhao Dai, Olivier Detante, Dominic Pérennou, Monica Baciu

**Affiliations:** a Univ. Grenoble Alpes, CNRS, LPNC, 38000 Grenoble, France; b Neurorehabilitation Department, Institute of Rehabilitation, Grenoble Alpes University Hospital, Grenoble, France; c Neurology Department, Grenoble Alpes University Hospital, Grenoble, France; d Grenoble Institute of Neurosciences, INSERM U1216, Grenoble Alpes University, La Tronche, France

**Keywords:** Aphasia severity, Outcome, Cognitive disorders, Support vector-regression symptom mapping, Lesion profile, Cognitive phenotype

## Abstract

In this study, we aimed to identify structural biomarkers linked to the severity and outcome of aphasia after a left-hemispheric stroke. We recruited 72 individuals with post-stroke aphasia and assessed their initial aphasia severity using the Aphasia Severity Rating Scale, alongside measures of naming ability and executive function. Aphasia outcome was determined for 56 out of 72 participants with available Aphasia Severity Rating Scale scores at discharge. We performed support-vector regression symptom mapping analyses at both cortical and white matter levels to examine the relationship between structural brain damage and our variables of interest (initial aphasia severity, naming, executive functions, and aphasia outcome). Our results revealed that (a) disconnections in white matter tracts within ventral and dorsal language pathways were associated with aphasia severity and naming deficits initially; and (b) disconnections in white matter tracts within executive networks (i.e., fronto-parietal, executive control, and salience networks) were related to executive dysfunction. This retrospective cohort study highlighted the crucial roles of white matter tracts within both dorsal (i.e., arcuate fasciculus, superior longitudinal fasciculus) and ventral (uncinate, inferior longitudinal, and middle longitudinal fasciculi) language streams in shaping the cognitive phenotypes of post-stroke aphasia patients, particularly concerning aphasia severity and naming impairment, by delineating distinct patterns of affected brain structures.

## Highlights

• Aphasia results from network disconnections.
• Dorsal and ventral language streams shape post-stroke aphasia cognitive phenotypes.
• Dorsal and ventral streams damage is linked to aphasia severity and naming deficits.
• Damage to executive control networks is related to executive dysfunction.

## 1 Introduction

Post-stroke aphasia is an acquired communication disorder that significantly impacts individuals’ quality of life, daily functioning, and engagement in family, social, and professional activities (Berg et al., 2022). Anomia, an impairment in finding words for objects, people, or actions, is the core feature of fluent and non-fluent aphasia (Kristinsson et al., 2021). During naming tasks, word retrieval requires (a) language-specific processes related to accessing, selecting, and manipulating semantic and phonological representations and (b) domain-general processes related to cognitive control and executive functions (EF) (Bontemps et al., 2024, Herbet and Duffau, 2020). Additionally, cognitive deficits, such as impairments in semantic memory, EF, and processing speed, often co-occur with aphasia and are associated with increased aphasia severity (Bonini et al., 2015, Fonseca et al., 2019). More specifically, executive dysfunction affects language abilities and recovery (Simic et al., 2019). Other factors, such as aphasia severity in acute and subacute phases (e.g., Lahiri et al., 2021, Pedersen et al., 2004) and lesion size and location (e.g., Na et al., 2022, Sul et al., 2019), also influence language recovery (for reviews, see Gerstenecker and Lazar, 2019, Watila and Balarabe, 2015).

Lesion-symptom mapping is used to investigate the relationship between brain lesions and resulting behavioral deficits. Two main methodological approaches are employed: univariate methods, such as voxel-based lesion-symptom mapping, and multivariate methods, such as multivariate lesion-symptom mapping. Bates and colleagues were among the first to use voxel-based lesion-symptom mapping to analyze the relationship between brain lesions and language abilities in individuals with post-stroke aphasia (Bates et al., 2003). Since then, other studies have employed lesion-symptom mapping to examine how brain damage relates to language performance, aphasia severity, and recovery (Baldo et al., 2011, Døli et al., 2021, Døli et al., 2023, Mirman et al., 2015, Park et al., 2021, Sul et al., 2019). For example, Døli et al. (2021) found that lesions in the left inferior frontal gyrus (IFG, *pars opercularis* and *triangularis*), insula, superior temporal gyrus (STG), and Heschl’s gyrus were associated with initial aphasia severity and deficits in reading, naming, and repetition. Sul et al. (2019) reported that lesions of the left precentral gyrus, Heschl’s gyrus, supramarginal cortex, STG and insula were related to a greater severity of aphasia one year post-stroke. Moreover, using multivariate lesion-symptom mapping, Levy et al. (2024) showed that more severe aphasia one year post-stroke was associated with damage to the posterior STG, precentral and middle frontal gyri, orbital gyrus, and basal ganglia. Finally, other studies showed an association between reduced speech fluency and damage to the rolandic operculum, middle frontal gyrus, temporal regions, precentral and postcentral gyri, putamen, IFG and insula (see Lacey et al., 2017, Yourganov et al., 2015).

Previous studies have also investigated the contribution of white matter (WM) tract damage to language disorders and recovery. Some focused on the integrity of WM tracts (i.e., tracts which overlap with the lesion; e.g., Basilakos et al., 2014, Kim et al., 2021, Sul et al., 2019), while others used indirect measures of WM tract disconnection (i.e., tracts which overlap with the lesion and those that extend beyond the lesion; e.g., Billot et al., 2022, Hope et al., 2016, Souter et al., 2022, Zavanone et al., 2018). This indirect measure represents an approach that infers structural disconnections in a patient by integrating spatial lesion data with anatomical information derived from healthy subjects (Sperber et al., 2022). For instance, Zavanone et al. (2018) reported that disconnections in the left uncinate fasciculus (UF) and inferior fronto-occipital fasciculus (IFOF) were related to the initial severity of aphasia, while disconnections in the left arcuate fasciculus (AF), UF, and IFOF were associated with poorer aphasia outcome at six months post-stroke. Another study showed that disconnections in the left AF and UF were associated with poorer verbal fluency and naming performance in chronic post-stroke aphasia (Hope et al., 2016). In our study, we defined disconnected WM tracts as in Billot et al. (2022), as combining lesion- and disconnectome-symptom mapping analyses offers additional insights into influence of both focal and distributed damage on behavioral deficits and outcomes.

Building upon previous research, it is crucial to understand how variations in lesion location relate to various degrees of language impairments (Na et al., 2022), especially considering the lack of consensus on factors influencing language outcomes (Kristinsson et al., 2022). Furthermore, given the established relationship between the initial severity of aphasia and aphasia outcome (El Hachioui et al., 2013, Kristinsson et al., 2022, Pedersen et al., 2004), it is essential to further investigate the factors that influence the initial severity of aphasia. In this context, given the need for clarification in the literature, the objective of our study is to explore how the location of damaged brain structures modulates, on the one hand, the initial severity of aphasia and, on the other hand, aphasia outcome at discharge from the rehabilitation ward. Additionally, taking into account the interaction between cognitive impairments and aphasia severity (Fonseca et al., 2019), and the fact that anomia is a core feature of aphasia (Kristinsson et al., 2021), we also investigate the impact of damaged brain structures on naming abilities and EF in individuals with post-stroke aphasia.

To this end, we examined the relationship between damaged brain regions or disconnected WM tracts and four key aspects: (i) initial aphasia severity (within 2 months post-stroke), (ii) naming deficits initially, (iii) initial EF deficits, and (iv) aphasia outcome at discharge (within 6 months post-stroke). We conducted separate symptom mapping analyses for each variable. Our objective was to pinpoint structural biomarkers of aphasia severity, aphasia-associated deficits and outcome in participants with left-hemispheric stroke. To achieve our goal, the originality of the method used is twofold: (i) we performed both support-vector regression lesion-symptom mapping (SVR-LSM) and disconnectome-symptom mapping (SVR-DSM) to explore the influence of focal and distributed damage, and (ii) we used a multimodal dataset that included lesion-related data and various cognitive measures to identify factors affecting not only aphasia outcome but also the initial severity and associated deficits during the acute and subacute phases post-stroke. Additionally, our study is a retrospective observational cohort study, which offers several advantages over randomized controlled trials, such as better generalizability by avoiding strict inclusion criteria that introduce sampling bias, a more cost-effective approach with less invasive or intensive data collection, and fewer ethical concerns as it does not involve experimental interventions (Bosdriesz et al., 2020).

## 2 Materials and methods

### 2.1 Study design and reporting

We conducted a monocentric observational study involving individuals with aphasia who experienced a left hemispheric stroke and were admitted to the neurological rehabilitation ward at University Hospital between January 2012 and October 2019. Each participant underwent two speech and language assessments: one at initial hospital admission (“first assessment”, FA) and another at discharge from the rehabilitation ward (“discharge assessment”, DA). A neuropsychological assessment was also conducted at FA. Sociodemographic, clinical, and neuroimaging data were collected from medical records. Our study follows the STROBE guidelines (see Supplementary Material, Table A).

### 2.2 Ethical and regulatory considerations

According to French law, observational studies involving retrospective data analysis are exempt from requiring approval from an ethics committee if participants are informed of the research and do not raise objections to using their data. All eligible participants were informed that their data would be used for research. Those who declined to participate signed an objection form.

### 2.3 Participants

The inclusion criteria were: age above 18 years, a left-hemispheric stroke confirmed by MRI, a diagnosis of aphasia confirmed by speech and language assessment, and a speech and language assessment performed at FA within 60 days post-stroke. Exclusion criteria were age over 80, recurrent strokes, complications in the acute stage (malignant infarct, cerebral herniation, subarachnoid haemorrhage, and hydrocephalus), dementia, unstable medical condition, severe psychiatric symptoms, inability to speak French, and lack of MRI data.

**Fig. 1:**
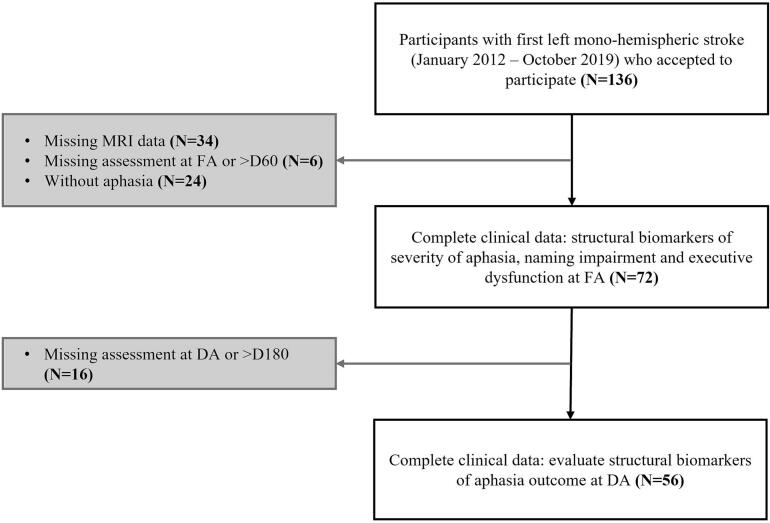
Participant Inclusion Flowchart in the Study.

A total of 136 individuals with a first left-hemispheric stroke were initially considered eligible and consented to participate in the study. Seventy-two participants were included in this study as they met the inclusion criteria (see Fig. 1).

### 2.4 Variables of interest: Initial severity of aphasia, naming, EF, and aphasia outcome

Based on the speech and language assessment performed at FA, we determined the initial severity of aphasia using the French version of Goodglass & Kaplan’s Aphasia Severity Rating Scale (ASRS), derived from the Boston Diagnostic Aphasia Examination (BDAE) (Goodglass and Kaplan, 1983, Mazaux and Orgogozo, 1981). The ASRS score, ranging from 0 to 5, indicates the degree of verbal communication abilities. A score of 0 means “no usable speech or auditory comprehension”, 1 corresponds to “all communication is through the fragmentary expression”, 2 to “conversation on familiar subjects is possible with help from listener”, 3 to “the patient can discuss almost all everyday problems with little or no assistance”, 4 to “some obvious loss of fluency or facility of comprehension without significant limitation on idea expressed”, and 5 to “minimal discernible speech handicaps” (Mazaux et al., 2013, Mazaux and Orgogozo, 1981). We also measured the delay between stroke onset and measurement of ASRS score at FA.

Naming abilities were assessed at FA using a picture naming task, either with the BDAE subtest (Mazaux & Orgogozo, 1981) or the DO80 (Deloche & Hannequin, 1997). To standardize results across participants who completed different naming tasks, we calculated a percentage of correct responses (ranging from 0 to 100 %) as a measure of naming performance. This approach also accounted for participants who experienced significant difficulties at FA and were unable to complete all items, resulting in variable maximum scores across participants. It should be noted that there was some missing data for naming score at FA (N = 11, 1.83 %).

EF were evaluated at FA with an ad-hoc executive scale based on several of the following tasks: Trail Making Test, Stroop, Wisconsin Card Sorting, verbal fluency (Godefroy & GREFEX, 2008), digit span and code subtests of the Wechsler Adult Intelligence Scale (WAIS-IV; Wechsler, 2011), tower of Hanoï or London (Coyette and Van der Linden, 1993, Culbertson and Zillmer, 2005, Simon, 1975), and Rey figure (Rey, 1959, Wallon and Mesmin, 2009). Due to the variability of the tasks performed by the participants (i.e., not all participants performed the same tasks), a neuropsychologist assigned an ad-hoc executive score ranging from 0 to 3, based on the results of the neuropsychological assessment. A score of 0 corresponding to “no impairment”, 1 to “mild”, 2 to “moderate”, and 3 to “severe” executive dysfunction.

Finally, individuals with aphasia underwent tailored speech and language therapy during their hospital stay. For participants whose speech and language assessment at DA was conducted within 180 days post-stroke (N = 56, see Fig. 1), we evaluated aphasia outcome in the late subacute phase using the ASRS score at DA. We considered that a low ASRS score at discharge, indicating more severe aphasia, reflected a poor aphasia outcome in this late subacute phase. For these 56 participants, we also measured (i) the intensity of speech and language therapy (average number of sessions per week), which was calculated based on the number of sessions and the duration of rehabilitation (in weeks); and (ii) the delay between stroke onset and measurement of ASRS score at DA.

### 2.5 Stroke-related factors: Damaged brain regions and disconnected WM tracts

#### 2.5.1 MRI Acquisition

An MR examination was performed two months post-stroke using a 1.5 T MR Magnetom Aera (Siemens, University Hospital). Three-dimensional gradient recalled echo T1-weighted images covering the whole brain were acquired for each participant (160 slices parallel to the bi-commissural plane, voxel size 0.9x0.9x0.9 mm, TR = 1.9 s, TE = 3.67 ms, flip angle = 15°). T2-Fluid Attenuated Inversion Recovery (FLAIR) images were also acquired: 30 slices parallel to the bi-commissural plane, slice thickness 4 mm, voxel size 0.7x0.7x4mm, TR = 9 s, TE = 74 ms.

#### 2.5.2 Lesion mapping

In each individual, lesions were manually delineated using MRIcron (https://www.nitrc.org/projects/mricron) from MRI axial slices (axial AC-PC, T2-FLAIR, 4 mm thickness). The lesion boundaries were manually and blindly delineated by a first trained investigator on axial slices of a T1-weighted MRI template from Montreal Neurological Institute (MNI) provided with MRIcron (ch2), then controlled by a second experienced investigator blind to the behavioral data. In case of disagreement on a lesion drawing, the different opinions were discussed until an agreement was reached. We did not use automatic segmentation and normalization algorithms on the MRIs performed in a clinical setting. Indeed, manual delineation is a reliable option for lesion analysis, particularly in elderly post-stroke participants, as age-related changes in cerebral architecture (e.g., ventricular dilatation or cerebral atrophy) can induce a mismatch between the injured areas and the template images used for automatic segmentation and normalization techniques (Kimberg et al., 2007, Rorden and Karnath, 2004).

The resulting 72 lesion drawings were used for overlap calculation indicating the number of individuals with a lesion in a given area using MRIcroGL software (https://www.nitrc.org/projects/mricrogl). Thus, at the group level, the “lesion overlay map” was obtained by overlaying patient’s lesion map on a template (mni152). This map represents the distribution of lesions in all participants.

#### 2.5.3 Disconnectome mapping

For each patient, a probability map of the WM tract’s disconnection was generated using the *Disconnectome map* tool within the BCB Toolkit software (Foulon et al., 2018). These maps provide an indirect estimate of the extent of structural disconnection at the voxel level. The estimate was obtained from the representations of WM fibers of healthy subjects that cross each lesion, called tractograms. The healthy subjects’ tractograms were obtained using a normative diffusion-weighted dataset from 163 healthy participants, acquired with 7 T MRI (Human Connectome Project; Vu et al., 2015). Thus, for each patient, the lesion map in MNI space was used to identify healthy controls’ WM fibers passing through the lesion. This process resulted in a percentage overlap map, which was generated by calculating the proportion of control participants whose tracts pass through the lesioned area, for each voxel in the MNI space. Consequently, in the resulting disconnectome map, in each voxel, the value takes into account the inter-individual variability of tract reconstruction within the dataset of healthy participants and shows a probability of disconnection, ranging from 0 % to 100 %, for a specific lesion. This disconnection probability is the voxel-wise probability of tracking a WM fiber of healthy participants entering the lesioned area when overlayed with the patient's lesion map. Each map encompasses (a) the WM tracts that overlap with the infarcted area and (b) the portion of WM tracts that extends beyond the infarcted area (Billot et al., 2022).

For each patient’s disconnectome map, a voxel is considered disconnected when the probability of disconnection is greater than 50 %, otherwise it is considered non-disconnected (Billot et al., 2022, Foulon et al., 2018). A “disconnectome overlay map” was then obtained, at a group level, by overlaying each patient’s disconnectome map on a template (mni152), using MRIcroGL software. This disconnectome overlay map represents the distribution of disconnected WM tracts in all participants.

### 2.6 Statistical analyses: support-vector regression symptom mapping

We aimed to evaluate the association between damaged brain structures (lesioned brain regions or disconnected WM tracts) and variables of interest (initial severity of aphasia, naming, EF, and aphasia outcome) through support-vector regression symptom mapping analyses. Indeed, we used a multivariate method based on a machine learning regression algorithm (i.e., support-vector regression) to investigate brain-behavior relationships.

To conduct these analyses, we used the *svrlsmgui* in MATLAB (version 2019b; DeMarco & Turkeltaub, 2018). Resub Loss method with 200 iterations was employed to determine the optimal hyperparameters related to our dataset (see Supplementary Material, Table B). Only voxels with damage in at least 10 % of the participants were included in each analysis, to avoid lowering statistical power by including infrequently damaged voxels (Sperber & Karnath, 2017). In our analyses, lesion maps serve as the input to SVR-LSM models, and disconnectome maps serve as the input to SVR-DSM models, and the four variables of interest were included as dependent variables. Moreover, as certain factors may influence behavioral scores (e.g., sex, age, stroke subtype, lesion volume, etc.), we performed additional statistical analyses to identify variables affecting behavioral performance (ASRS at FA and DA, naming, and EF). Using *svrlsmgui*, covariates can be regressed out of the lesion data, the behavioral score, or both. Based on the results of these additional statistical analyses (see Supplementary Material, Table C) and considering the relationship between lesion volume and local lesion status, the following covariate regressions were applied: (i) regression of lesion volume out of both the ASRS score at FA and the lesion maps; (ii) regression of lesion volume out of both the naming score and the lesion maps, and regression of age out of the naming score; (iii) regression of age and stroke subtype out of the EF score, and regression of lesion volume out of the lesion maps; and (iv) regression of the ASRS score at FA out of aphasia severity at discharge, and regression of lesion volume out of the lesion maps. The output of SVR-symptom mapping analyses are β-values, which represent the strength of the relationship between the lesion/disconnection status for each voxel and the behavior. The SVR-β maps were generated using 5-fold cross-validation. The resulting SVR-β values were thresholded voxel-wise at p < 0.005 and corrected for cluster size at p < 0.05, both based on 5,000 permutations. Based on these SVR-symptom mapping analyses, we presented either voxel-wise or cluster-wise thresholded results to show the association between damaged brain structures and behavioral scores. Specifically, voxel-wise thresholded results were presented when no clusters survived the cluster-wise correction; otherwise, cluster-wise results were reported.

Initially, all 72 participants were included in the analyses of initial severity of aphasia, naming, and EF. For aphasia outcome at discharge, only data from 56 participants who underwent a speech and language assessment at both FA and DA were included. However, some participants were excluded from the analyses due to missing behavioral data or for having no voxels inside the minimum lesion overlap cutoff. Therefore, SVR-LSM analyses were conducted on: (i) 70 participants for initial severity of aphasia and EF, (ii) 59 for naming deficits; and (iii) 54 for aphasia outcome at discharge. For SVR-DSM analyses, the relationship between disconnected WM tracts and naming was tested in 61 participants, and no exclusions were necessary for the remaining variables (i.e., initial severity, EF, and aphasia outcome).

The AAL (Tzourio-Mazoyer et al., 2002) and HCP1065 (Yeh, 2022) atlases were used to identify structures highlighted by the SVR-LSM and SVR-DSM analyses, respectively. For SVR-DSM analyses, WM tracts were separated according to their role: association, projection, and commissural. Using SVR-LSM and SVR-DSM analyses, we identified clusters associated with the variables of interest, along with the identified regions or WM tracts within each cluster and the number of voxels for each WM tract (measured separately for each pathway category: association, projection and commissural) or each region. All results were visualized using MRIcroGL software.

## 3 Results

### 3.1 Participants

Seventy-two individuals with aphasia who experienced a left-hemispheric stroke and satisfied the inclusion criteria were included in this study (see Fig. 1). Sociodemographic and clinical profiles for these participants are available in the Supplementary Material, Table D.

The median age of all participants was 64.11 years (interquartile range Q1-Q3 = 54.12–70.72). Among them, 47 (65.28 %) were males, and 68 (94.44 %) were right-handed. The majority of participants had an ischemic stroke (79.17 %), and the median size of the lesion was 21,732 voxels (7,386–48,090). The median severity of aphasia at FA was 2 (1–3), and the median accuracy in naming was 70.00 % (18.75–85.00). Additionally, participants exhibited moderate executive dysfunction, evidenced by a median score of 2 (1–2). Finally, the median delay between stroke onset and measurement of ASRS score at FA was 19.00 days post-stroke (11.75–26.50).

**Fig. 2:**
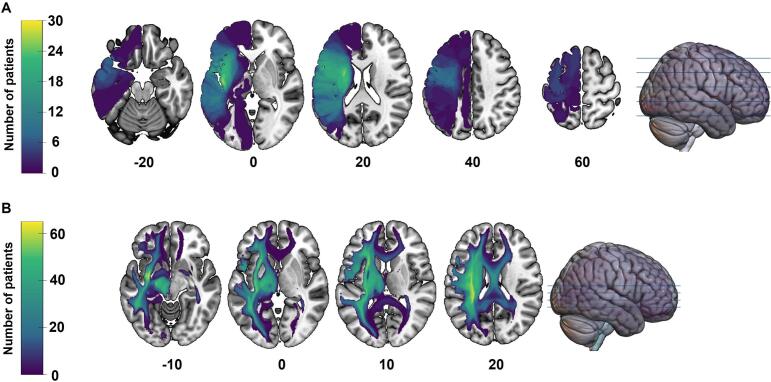
Lesion (A) and disconnectome (B) overlay maps for all participants (N = 72). Axial slices are displayed with the left hemisphere on the left side, and z coordinates in MNI space are indicated below each slice. Lighter shades (green and yellow) represent (A) the injured voxels shared by the largest number of participants and (B) voxels with a higher prevalence of disconnection among participants. (For interpretation of the references to colour in this figure legend, the reader is referred to the web version of this article.)

The lesion overlay map revealed that the areas with the highest degree of overlap among participants included the left insula, rolandic operculum, IFG *pars opercularis* and *pars triangularis*, putamen, precentral and postcentral gyri, Heschl’s gyrus, STG, and caudate nucleus (see Fig. 2A). The disconnectome map revealed that the WM tracts with the highest overlay among all participants encompassed the left IFOF, AF, frontal aslant tract (FAT), superior longitudinal fasciculus (SLF), extreme capsule, inferior longitudinal fasciculus, thalamic radiations, and left-sided cortico-spinal, cortico-striatal, reticulo-spinal, cortico-pontine, dentato-rubro-thalamic tracts, medial lemniscus, fornix, optic and acoustic radiations, along with the corpus callosum (CC) and anterior commissure bilaterally (see Fig. 2B).

Additionally, aphasia outcome at discharge was evaluated in 56 of the 72 participants initially screened, since this evaluation specifically focused on individuals with aphasia who underwent a speech and language assessment at both FA and DA. For these 56 participants, the median intensity of speech and language therapy was 4.40 days per week (2.23–5.00), and the median delay between stroke onset and measurement of ASRS score at DA was 85.50 days post-stroke (62.00–123.50).

### 3.2 Support-vector regression lesion-symptom mapping (SVR-LSM)

**Fig. 3:**
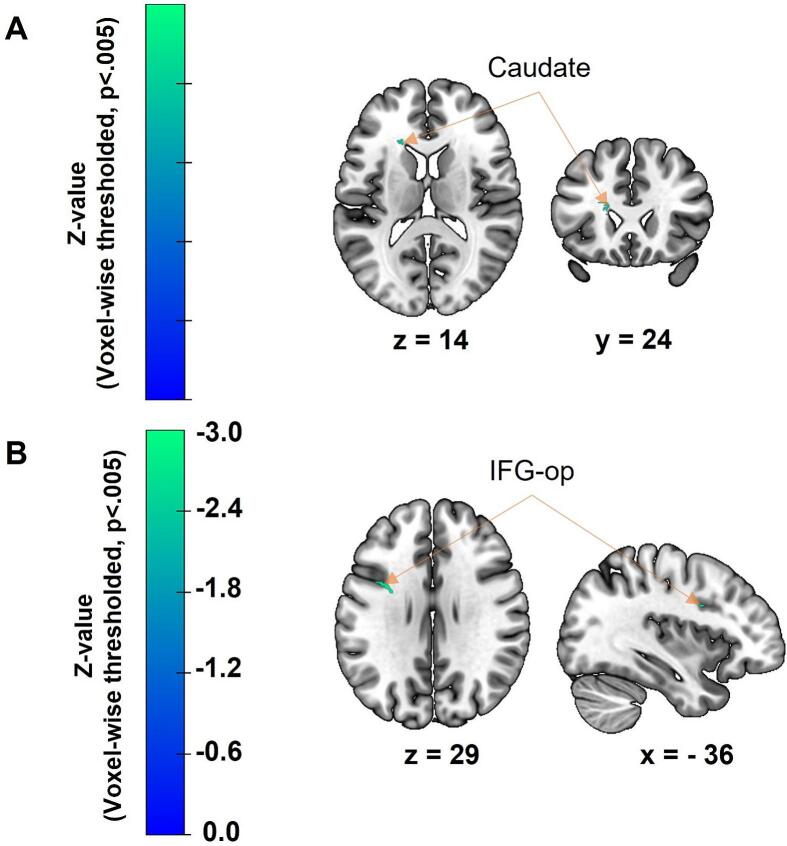
Results of SVR-LSM analyses showing the association between gray matter lesions and behavioral scores, namely (A) executive dysfunction initially, and (B) aphasia outcome at discharge. Voxel-wise thresholding was applied (p < 0.005, 5,000 permutations). Presented are sagittal, coronal, and/or axial slices, with x, y, or z coordinates in MNI space indicated below each slice. More negative z-values (i.e., colors shifting towards green) reflect a stronger link between lesion status and behavioral scores. *IFG-op, Inferior Frontal Gyrus pars opercularis.* (For interpretation of the references to colour in this figure legend, the reader is referred to the web version of this article.)

Using SVR-LSM analyses, we aimed to identify damaged brain regions associated with each of the four key variables: the initial severity of aphasia, naming deficits, executive dysfunction, and aphasia outcome at discharge. The brain regions identified by SVR-LSM analyses are detailed in Fig. 3 and Table 1. Considering that no cluster-wise results survived thresholding (p < 0.05, 5,000 permutations), we presented voxel-wise thresholded results at p < 0.005 based on 5,000 permutations.

**Table 1:** Damaged brain regions associated with executive dysfunction and aphasia outcome at discharge, identified using the AAL atlas and based on SVR-LSM analyses. Voxel-wise thresholded results (p < 0.005, 5,000 permutations) are presented. Identified regions are located in the left hemisphere and constitute several clusters.

**Executive dysfunction at FA**	**Total volume (in voxels)**	**155**	**71**

	**Peak z-values**	**−3.3**	**−3.0**

	**Peak X Y Z**	**−19.0 x 25.7 x 16.0**	**−29.3 x −47.3 x 22.6**

Left Caudate	29	−

Unidentified	126	71

**Aphasia outcome at discharge**	**Total volume (in voxels)**	**61**	

	**Peak z-values**	**−2.9**	

	**Peak X Y Z**	**−30.0 x −0.1 x 29.3**	

Left Inferior Frontal Gyrus, *pars opercularis*	32	

Unidentified	29	


We present identified regions within the cluster and the number of voxels for each region. Some “unidentified” voxels are not categorized according to the AAL atlas.

Regarding the initial severity of aphasia and naming abilities, the SVR-LSM analyses did not reveal any clusters that survived either voxel-wise or cluster-wise thresholding.

Additionally, we identified two clusters (155 and 71 voxels) associated with greater EF deficits. These clusters were significant after voxel-wise thresholding, with z-values between 0 and −3.3 (see Fig. 3A and Table 1). The largest cluster (155 voxels) involved voxels in the left caudate (29 voxels). The smallest cluster (71 voxels) was not discussed as it included voxels outside the gray matter regions defined in the AAL atlas.

Regarding aphasia outcome at discharge, one small cluster (61 voxels) survived voxel-wise thresholding, with z-values ranging from 0 to −2.9 (see Fig. 3B and Table 1). This cluster involved voxels in the left IFG *pars opercularis* (IFG-op, 32 voxels).

### 3.3 Support-vector regression disconnectome-symptom mapping (SVR-DSM)

**Fig. 4:**
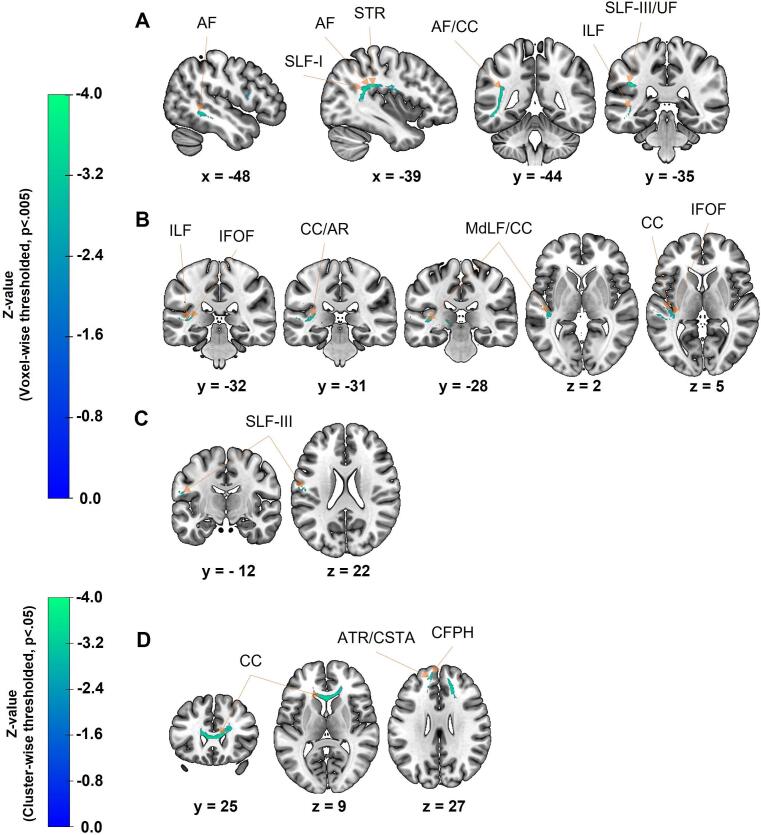
Results of SVR-DSM analyses showing the association between disconnected WM tracts and behavioral scores, namely (A) initial severity of aphasia, (B) naming impairment initially, (C) aphasia outcome at discharge, and (D) executive dysfunction initially. Voxel-wise thresholding was applied (p < 0.005, 5,000 permutations) except for executive dysfunction initially, which uses cluster-wise thresholding (p < 0.05, 5,000 permutations). Only WM tracts within clusters that contained more than 100 significant voxels are labeled. Presented are sagittal, coronal, and/or axial slices, with x, y, or z coordinates in MNI space indicated below each slice. More negative z-values (i.e., colors shifting towards green) indicate a stronger link between WM disconnections and behavioral scores. *AF, Arcuate Fasciculus; AR, Acoustic Radiation; ATR, Anterior Thalamic Radiation; CC, Corpus Callosum; CFPH, Cingulum Frontal Parahippocampal; CSTA, Corticostriatal Tract Anterior; IFOF, Inferior Fronto-Occipital Fasciculus; ILF, Inferior Longitudinal Fasciculus; MdLF, Middle Longitudinal Fasciculus; SLF, Superior Longitudinal Fasciculus; STR, Superior Thalamic Radiation; UF, Uncinate Fasciculus.* (For interpretation of the references to colour in this figure legend, the reader is referred to the web version of this article.)

Considering SVR-DSM analyses, we identified disconnected WM tracts associated with the four variables of interest (see Fig. 4 and Table 2). We report only voxel-wise thresholded results (p < 0.005, 5,000 permutations), except for EF, for which cluster-wise thresholded results are presented (p < 0.05, 5,000 permutations). Additionally, we detailed only identified clusters that contained more than 100 significant voxels after voxel-wise or cluster-wise thresholding.

**Table 2:** Disconnected WM tracts associated with the initial severity of aphasia, executive dysfunctions at the first assessment (FA), naming deficits at FA, and aphasia outcome at discharge, identified using SVR-DSM analyses with the HCP1065 atlas. Results are voxel-wise thresholded (p < 0.005, 5,000 permutations), except for executive dysfunction, which uses cluster-wise thresholding (p < 0.05, 5,000 permutations). Tracts are in the left hemisphere, except for commissural pathways.

		Initial severity of aphasia	Executive dysfunction at FA	Naming deficits at FA	Aphasia outcome at discharge
	**Total volume (in voxels)**	**1,644**	**5,869**	**389**	**117**
	**Peak z-values**	**−3.5**	**−3.5**	**−3.4**	**−3.1**
	**Peak X Y Z**	**−38.9 x −37.0 x 25.6**	**17.9 x 31.6 x 10.1**	**−35.9 x −30.4 x 4.9**	**−58.8 x −14.9 x 21.9**
**Left Association Pathways**	Arcuate Fasciculus	641	−	−	−

	Superior Longitudinal Fasciculus I	82	−	−	−

	Inferior Longitudinal Fasciculus	49	−	23	−

	Superior Longitudinal Fasciculus III	49	−	4	40

Uncinate Fasciculus	33	−	−	−

Cingulum Frontal Parahippocampal	−	822	−	−

Middle Longitudinal Fasciculus	−	−	179	−

**Left Projection Pathways**	Superior Thalamic Radiation	49	−	−	−

	Corticostriatal Tract Anterior	−	176	−	−

Anterior Thalamic Radiation	−	117	−	−

Acoustic Radiation	−	−	82	

**Commissural Pathways**	Corpus callosum	1,282	3,404	222	−

We present identified WM tracts within the clusters and the number of voxels for each WM tract, measured separately in each pathway category (association, projection and commissural). Some voxels are not categorized according to the HCP1065 atlas (‘unidentified’) and have not been included to improve table readability. Identified clusters with fewer than 100 voxels are not displayed, as they have not been discussed.

Regarding the initial severity of aphasia, we found three significant clusters after voxel-wise thresholding (1,644, 83, 68 voxels), with z-values ranging from 0 to −3.5 (see Fig. 4A and Table 2). The largest cluster (1,644 voxels) included voxels in the left AF (641 voxels) and the CC bilaterally (1,282 voxels). Other WM tracts containing a smaller number of voxels were also identified, including the left SLF-I (82 voxels), inferior longitudinal fasciculus (ILF, 49 voxels), SLF-III (49 voxels), UF (33 voxels), superior thalamic radiations (STR, 49 voxels). The smallest clusters (83 and 68 voxels) were not presented and not discussed due to their limited contribution (<100 voxels).

We identified four clusters (from 32 to 389 voxels) associated with reduced naming abilities, with z-values ranging from 0 to −3.4 after voxel-wise thresholding (see Fig. 4B and Table 2). The largest cluster (389 voxels) included voxels in the CC bilaterally (222 voxels) and the left middle longitudinal fasciculus (MdLF, 179 voxels). Other WM tracts containing a smaller number of voxels were also identified, including the left ILF (23 voxels), SLF-III (4 voxels), and acoustic radiation (82 voxels). The smallest clusters (32, 39, and 55 voxels) were not presented and not discussed due to their limited contribution (<100 voxels).

Moreover, we identified a cluster (117 voxels) associated with more severe aphasia at discharge, suggesting poor aphasia outcome (see Fig. 4C and Table 2). This cluster was significant after voxel-wise thresholding, with z-values ranging from 0 to −3.1. The cluster included voxels in the left SLF-III (40 voxels).

Finally concerning EF, we identified a cluster encompassing 5,869 voxels associated with more severe executive dysfunction initially (see Fig. 4D and Table 2). This cluster survived cluster-wise thresholding, with z-values ranging from 0 to −3.5. It included voxels in the left frontal parahippocampal segment of the cingulum (CFPH, 822 voxels), anterior cortico-striatal tract (176 voxels), anterior thalamic radiations (ATR, 117 voxels), and the CC bilaterally (3,404 voxels).

## 4 Discussion

In this study, we aimed to identify structural biomarkers of aphasia severity, aphasia-associated deficits and outcome in individuals with post-stroke aphasia using SVR-symptom mapping analyses. Our findings showed that disconnections in the CC and the left AF, as well as to a lesser extent SLF-I/III, ILF, UF and STR, were associated with higher severity of aphasia initially. Moreover, we showed that damage to the left caudate, and disconnections in the left frontal parahippocampal segment of the cingulum (CFPH), ATR, anterior corticostriatal tract (CST), and the CC bilaterally were associated with more severe executive dysfunction initially. Additionally, we showed that reduced naming abilities initially were associated with disconnections in the left MdLF and the CC bilaterally, as well as to a lesser extent the left ILF, SLF-III, and acoustic radiations. Finally, damage to the left IFG-op and disconnections in the left SLF-III were associated with poor aphasia outcome at discharge.

In the following paragraphs, we will discuss the results for each of the four analyzed factors separately, focusing on cortical lesions and disconnected WM tracts. More specifically, some of the SVR-symptom mapping analyses revealed poor predictive performance, with negligible prediction accuracy (i.e., correlation coefficients between predicted and true values not statistically significant, with values ranging from −0.2 to 0.2; see Supplementary Material, Table B and Figure A). Moreover, a discrepancy exists between prediction and brain mapping: the presence of a statistically significant association does not necessarily imply predictive value (Sperber et al., 2025). Consequently, our results are interpreted as statistical neural correlates, i.e., associations between disconnected WM tracts and behavioral scores, rather than as predictions of these scores.

First, regarding the relationship between our variables of interest (i.e., aphasia severity at FA and DA, naming score, and EF score) and damaged brain regions, no significant associations were identified between these variables and cortical regions. When clusters were identified, they were negligible in extent (i.e., identified regions comprising fewer than 50 voxels). This lack of significant results may reflect the combined influence of a relatively small sample size (<100 participants) and the rigorous analytical approach employed (SVR-symptom mapping with hyperparameter optimization and cross-validation), which, although enhancing methodological robustness, may limit the detection of weaker or more spatially localized effects.

Moreover, based on SVR-DSM analyses, we discuss the relationship between ***initial aphasia severity*** and disconnected WM tracts. Aphasia severity was assessed using the ASRS score, which primarily measures impairments in oral expression based on open-ended conversation and spontaneous speech tasks (Mazaux & Orgogozo, 1981). Our results suggest that disconnections in the CC and the left AF, as well as to a lesser extent the left SLF-I/III, ILF, UF, and STR, were associated with more severe aphasia initially. Kim et al. (2021) showed that lesions of the left AF, SLF, corona radiata in the frontal and temporal lobes, and the CC were associated with higher initial severity of aphasia. Other studies have reported that disconnections in the left UF, IFOF (Zavanone et al., 2018), anterior segment of the AF, and FAT (Forkel & Catani, 2018) were associated with more severe aphasia at baseline. While our findings are partly consistent with these studies, discrepancies may be explained by methodological differences in assessing aphasia severity and WM tract damage. The identified WM tracts are critically involved in speech and language processes, which likely explains their association with aphasia severity (Basilakos et al., 2014, Cristofori et al., 2015, Hope et al., 2016, Kim et al., 2021, Monroy-Sosa et al., 2021, Ribeiro et al., 2024). Specifically, the left AF and SLF are part of the dorsal phonological stream, while the ILF and UF belongs to the ventral stream (Duffau et al., 2014). The AF supports speech fluency, and phonological and complex syntactic processing during both language production and comprehension (Basilakos et al., 2014, Dick et al., 2014). The left SLF-I is involved in verbal working memory, while the left SLF-III contributes to articulatory aspects of language and phonological loop in working memory (Monroy-Sosa et al., 2021, Ribeiro et al., 2024). The ILF and UF are part of the ventral stream and play a role in semantic processing and comprehension (Catani et al., 2013, Jarret et al., 2022). The STR, which constitutes the medial part of the superior corona radiata (Younes et al., 2019), is involved in verbal fluency (Cristofori et al., 2015), consistent with the association between the ASRS score and speech fluency (Mazaux & Orgogozo, 1981). Finally, the posterior part of the corpus callosum plays a role in phonological and semantic processing during naming (Hula et al., 2020). Therefore, disconnections in these WM tracts, including both ventral and dorsal language pathways, may disrupt the neural networks critical for speech and language processing, contributing to more severe aphasia.

With regard to ***executive dysfunction***, our findings indicate that disconnections in the CC, left CFPH, ATR, and anterior CST were associated with more severe executive dysfunction. These findings align with evidence supporting the role of the anterior CC in working memory tasks, and the involvement of the cingulum bundle in EF within a large fronto-parietal network. Specifically, the anterior, posterior and left parahippocampal segments of the cingulum are involved in verbal working memory (Ribeiro et al., 2024). Additionally, several studies showed that the integrity of CST and cortico-thalamic tract is associated with executive functioning (see Ferris et al., 2022, Ribeiro et al., 2024). Furthermore, damage to the left fronto-striatal tract, fronto-parietal tract, and FAT was associated with global executive dysfunction in individuals with surgically resected low-grade gliomas (Cochereau et al., 2020). Our results are consistent with the role of the CC, left CFPH, ATR, and anterior CST in EF. However, to better understand the relationship between executive dysfunction and damaged brain structures, future studies should employ more detailed and formal assessments of EF, using the same standardized tests in all participants that separately measure core executive components, rather than a global ad-hoc executive score.

Regarding ***naming abilities,*** we found that disconnections in the left MdLF and the CC bilaterally, and to a lesser extent the left ILF, SLF-III, and acoustic radiations. Hula et al. (2020) found that the integrity of the left AF, MdLF, UF, IFOF was associated with semantic processing during picture naming, while the left AF and MdLF were associated with phonological processing. Another study showed that naming abilities were associated with the integrity of the left IFOF, AF, UF, and forceps minor of the CC (Han et al., 2024). Our findings showed that WM tracts involved in both the dorsal and ventral language streams were associated with naming abilities. Nevertheless, naming abilities were primarily associated with integrity of WM tracts involved in the ventral stream (MdLF and ILF), while the dorsal stream (SLF) demonstrated only a minimal implication. The left MdLF is involved in both semantic and phonological processing during picture naming (Duffau et al., 2014, Hula et al., 2020, Monroy-Sosa et al., 2021). Additionally, the left SLF-III and the CC contribute to working memory and EF, both of which are required during naming tasks (Bontemps et al., 2024, Monroy-Sosa et al., 2021, Ribeiro et al., 2024). The posterior part of the CC is also involved in phonological and semantic naming functions (Hula et al., 2020). Moreover, the ILF is involved in picture naming, as it plays a role in lexical retrieval for visual stimuli (Papagno et al., 2023). Finally, the acoustic radiations, as a thalamo-cortical projection, are involved in speech processing and music perception (Maffei et al., 2018).

Finally, we discuss the relationship between ***aphasia outcome at discharge*** and disconnected WM tracts. Our findings suggest that disconnections in the left SLF-III were associated with poor aphasia outcome at discharge. Previous studies have reported that disconnections in the left UF, IFOF, and AF were associated with poor outcome six months post-stroke (Zavanone et al., 2018), and more severe aphasia in chronic phase was associated with lesions of the left posterior corona radiata, SLF (Sul et al., 2019), AF, and IFOF (Rosso et al., 2015). In our study, we found different results which may be related to the method used to assess aphasia outcome at discharge. Indeed, we considered that more severe aphasia at discharge from the rehabilitation ward indicated poor aphasia outcome in the late subacute phase. As mentioned previously, the left SLF-III contributes to verbal working memory and articulatory aspects of language (Monroy-Sosa et al., 2021, Ribeiro et al., 2024). However, it should be noted that the SLF-III demonstrated only a minimal implication in our results.

In this study, we aimed to identify how profiles of impacted brain structures relate to the cognitive characteristics of participants with aphasia following a left-hemispheric stroke. A key contribution of our study is the use of a multimodal approach, combining structural neuroimaging (i.e., grey matter lesions and WM tract disconnections) with cognitive and language assessments. Using symptom mapping analyses, we were able to pinpoint structural biomarkers associated with cognitive phenotypes of participants with post-stroke aphasia, defined by aphasia severity and associated deficits. Our findings revealed that: (a) disconnections in WM tracts of both ventral and dorsal language pathways were associated with aphasia severity and naming abilities initially; and (b) integrity of WM tracts within executive networks (including fronto-parietal, executive control, and salience networks) was associated with better executive functioning. Specifically, damage to the left AF, SLF (involved in the dorsal stream) and the left ILF, UF (involved in the ventral stream) were associated with more severe aphasia initially. Naming deficits were associated with damage to the left SLF (dorsal stream), and ILF and MdLF (ventral stream). These findings underline the critical role of both dorsal and ventral language streams in language processes, as damage to these pathways correlates with more severe aphasia and naming deficits, consistent with prior studies (e.g., see Fridriksson et al., 2018, Zhang et al., 2018). Finally, EF impairments were associated with damage to executive control, fronto-parietal and salience networks, which include the left CFPH, ATR, and anterior CST.

This study has several limitations. First, the sample size for investigating the damaged brain structures associated with aphasia outcome or naming was relatively small. In addition, some participants were excluded from the analyses due to missing behavioral data or insufficient lesion overlap. Therefore, replication in larger and more representative cohort is needed to validate our findings. Second, EF was assessed using an ad-hoc executive score that combines several executive skills. A more detailed assessment with raw scores could better isolate brain structures associated with specific EF components. Third, two different tasks (DO80 and BDAE subtask) were used to evaluate naming abilities. To standardize results across participants who completed different naming tasks with different maximum scores, we calculated a percentage of correct responses. Nevertheless, using raw scores from a single task might be more appropriate in future studies to avoid potential variability in item difficulty or number. Fourth, aphasia severity was measured using the ASRS score, which primarily reflects oral expression through speech production tasks. Future research should consider more comprehensive scores or tools assessing both expression and comprehension.

Finally, in the present study, which involved a relatively small sample derived from retrospective data and focused on a limited set of factors (i.e., severity of aphasia and associated deficits) with selected covariates, SVR-symptom mapping analyses proved to be computationally demanding and time-consuming. Consequently, to better understand the factors influencing aphasia severity, associated deficits, and outcome, future studies with larger cohorts (>100–150 participants) should adopt a more holistic approach. Such studies should consider not only damaged brain structures but also sociodemographic (e.g., sex, age, handedness), cognitive and clinical (e.g., general disability, apraxia, mood disorders), rehabilitation-related (e.g., duration and intensity of speech and language therapy), and stroke-related (e.g., lesion size, stroke severity, type of stroke, type of aphasia) factors. Moreover, future research should aim to identify network-based biomarkers (i.e., affected regions or disconnected WM tracts associated with post-stroke language and cognitive impairments), using longitudinal designs that extend beyond the subacute phase, in order to capture and predict their impact on long-term recovery and functional outcomes.

## 5 Conclusion

In this retrospective observational study, we aimed to identify structural biomarkers of aphasia-associated deficits and outcome in individuals with aphasia following left-hemispheric stroke. Our results reveal distinct profiles of WM damage that underlie initial severity of aphasia, naming deficits, executive dysfunction, and aphasia outcome. We specifically underscore the key and complementary roles of dorsal and ventral language streams in language abilities. Importantly, our findings demonstrate that aphasia arises from disconnections within distributed neural networks, as revealed by WM tract disconnection analyses. This emphasizes the value of using multimodal approach, combining structural neuroimaging with language and neuropsychological assessments, to map the substrates underlying post-stroke language and cognitive impairments. Considering the poor prediction performance in this study, future research should further investigate these network-based biomarkers in larger longitudinal cohorts to enhance clinical care and optimize recovery for individuals with post-stroke aphasia.

## CRediT authorship contribution statement

**Célise Haldin:** Writing – review & editing, Writing – original draft, Visualization, Methodology, Investigation, Formal analysis, Data curation, Conceptualization. **Hélène Lœvenbruck:** Writing – review & editing, Writing – original draft, Supervision, Resources, Methodology, Conceptualization. **Céline Piscicelli:** Writing – review & editing, Resources, Methodology, Investigation, Data curation, Conceptualization. **Valérie Marcon:** Writing – review & editing, Resources, Methodology, Investigation, Data curation, Conceptualization. **Shenhao Dai:** Writing – review & editing, Writing – original draft, Visualization, Resources, Methodology, Investigation, Formal analysis, Data curation, Conceptualization. **Olivier Detante:** Writing – review & editing, Resources, Data curation, Conceptualization. **Dominic Pérennou:** Writing – review & editing, Writing – original draft, Supervision, Resources, Project administration, Methodology, Conceptualization. **Monica Baciu:** Writing – review & editing, Writing – original draft, Supervision, Resources, Project administration, Methodology, Conceptualization.

## Funding

This work has been supported by the ANR project ANR-15-IDEX-02, by the Labex CerCoG and the Research Proposal IRGA funded by the Université Grenoble Alpes.

## Declaration of competing interest

The authors declare that they have no known competing financial interests or personal relationships that could have appeared to influence the work reported in this paper.

## Data Availability

As the data presented in this article consist of patient health information, and in accordance with French regulations and ethical standards, we are unable to make the dataset publicly available. However, we can share the analysis pipelines used in this study upon request.
