# Supplementary figures and images for: Structural correlates of aphasia severity, cognitive impairment, and outcome after stroke
Source: Neuroimage Clin. 2026 Jan 19;49:103954. doi: 10.1016/j.nicl.2026.103954 (PMC12874455; doi:10.1016/j.nicl.2026.103954)

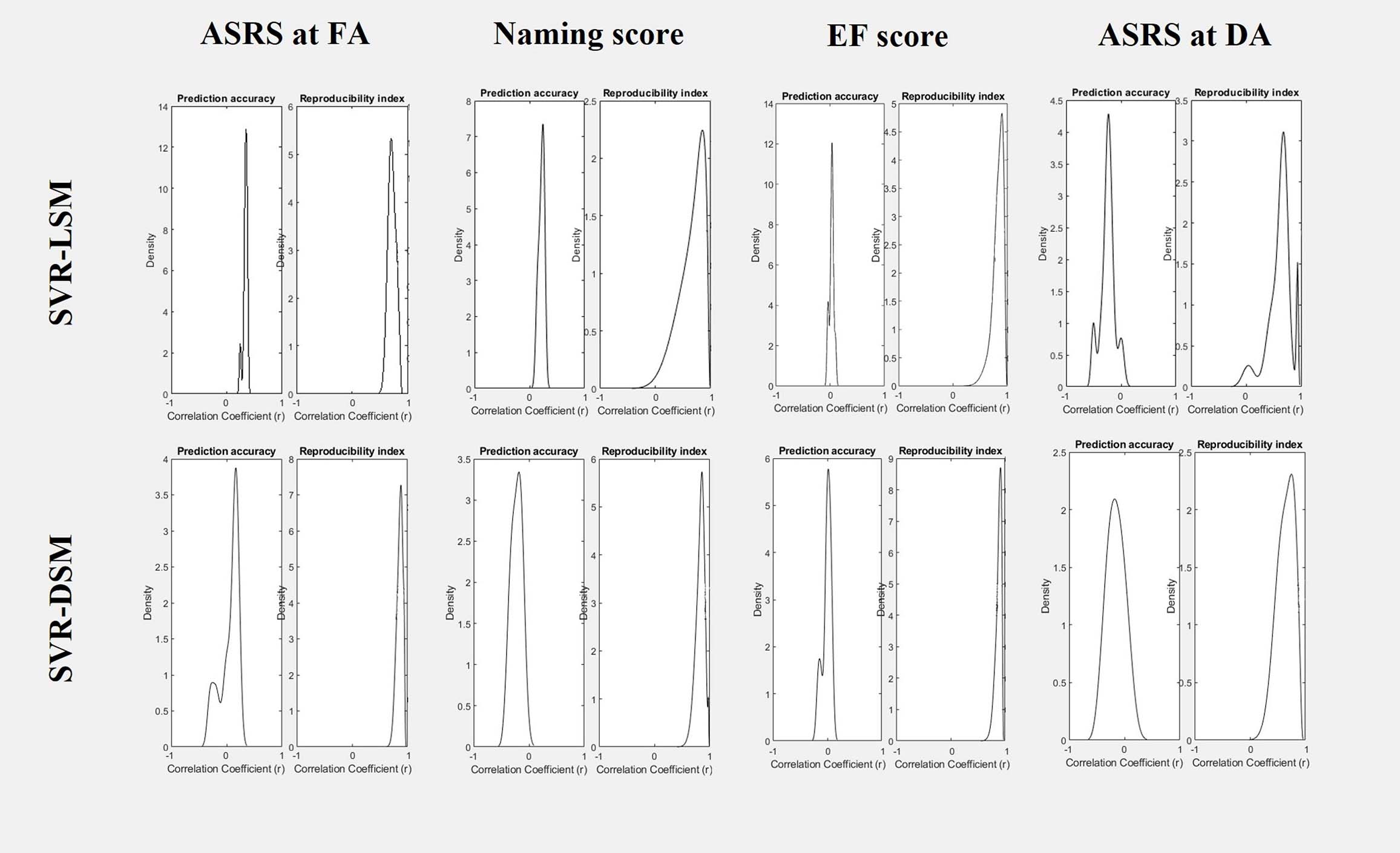

Supplement: Supplementary Figure 1 [file mmc1.jpg]
