# Supplementary material for: Structural correlates of aphasia severity, cognitive impairment, and outcome after stroke
Source: Neuroimage Clin. 2026 Jan 19;49:103954. doi: 10.1016/j.nicl.2026.103954 (PMC12874455; doi:10.1016/j.nicl.2026.103954)
Supplement: Supplementary Data 2 [file mmc2.docx]

**Table A.** STROBE Statement. Checklist of items that should be included in reports of cohort studies.

|  | | Item No | Recommendation | Page No |
| --- | --- | --- | --- | --- |
| **Title and abstract** | | 1 | (*a*) Indicate the study’s design with a commonly used term in the title or the abstract | 1-2 |
|  |  |  | (*b*) Provide in the abstract an informative and balanced summary of what was done and what was found | 2 |
| Introduction | | | | |
| Background/rationale | | 2 | Explain the scientific background and rationale for the investigation being reported | 4-5 |
| Objectives | | 3 | State specific objectives, including any prespecified hypotheses | 6-7 |
| Methods | | | | |
| Study design | | 4 | Present key elements of study design early in the paper | 7 |
| Setting | | 5 | Describe the setting, locations, and relevant dates, including periods of recruitment, exposure, follow-up, and data collection | 7 |
| Participants | | 6 | (*a*) Give the eligibility criteria, and the sources and methods of selection of participants. Describe methods of follow-up | 7-8 |
|  |  |  | (*b*) For matched studies, give matching criteria and number of exposed and unexposed | NA |
| Variables | | 7 | Clearly define all outcomes, exposures, predictors, potential confounders, and effect modifiers. Give diagnostic criteria, if applicable | 8-11 |
| Data sources/ measurement | | 8* | For each variable of interest, give sources of data and details of methods of assessment (measurement). Describe comparability of assessment methods if there is more than one group | 8-11 |
| Bias | | 9 | Describe any efforts to address potential sources of bias | 11-13 |
| Study size | | 10 | Explain how the study size was arrived at | 8-9, 13 |
| Quantitative variables | | 11 | Explain how quantitative variables were handled in the analyses. If applicable, describe which groupings were chosen and why | 8-11 |
| Statistical methods | | 12 | (*a*) Describe all statistical methods, including those used to control for confounding | 11-13 |
|  |  |  | (*b*) Describe any methods used to examine subgroups and interactions | NA |
|  |  |  | (*c*) Explain how missing data were addressed | 13 |
|  |  |  | (*d*) If applicable, explain how loss to follow-up was addressed | NA |
|  |  |  | (*e*) Describe any sensitivity analyses | NA |
| Results | | | |  |
| Participants | | 13* | (a) Report numbers of individuals at each stage of study—eg numbers potentially eligible, examined for eligibility, confirmed eligible, included in the study, completing follow-up, and analysed | 14-15 |
|  |  |  | (b) Give reasons for non-participation at each stage | 13 |
|  |  |  | (c) Consider use of a flow diagram | 14 |
| Descriptive data | | 14* | (a) Give characteristics of study participants (eg demographic, clinical, social) and information on exposures and potential confounders | 14-15 |
|  |  |  | (b) Indicate number of participants with missing data for each variable of interest | 8, Table D |
|  |  |  | (c) Summarise follow-up time (eg, average and total amount) | NA |
| Outcome data | | 15* | Report numbers of outcome events or summary measures over time | 14-15 |
| Main results | 16 | (*a*) Give unadjusted estimates and, if applicable, confounder-adjusted estimates and their precision (eg, 95% confidence interval). Make clear which confounders were adjusted for and why they were included | | 15-17 |
|  |  | (*b*) Report category boundaries when continuous variables were categorized | | NA |
|  |  | (*c*) If relevant, consider translating estimates of relative risk into absolute risk for a meaningful time period | | NA |
| Other analyses | 17 | Report other analyses done – e.g., analyses of subgroups and interactions, and sensitivity analyses | | NA |
| Discussion | | | | |
| Key results | 18 | Summarise key results with reference to study objectives | | 17 |
| Limitations | 19 | Discuss limitations of the study, taking into account sources of potential bias or imprecision. Discuss both direction and magnitude of any potential bias | | 22 |
| Interpretation | 20 | Give a cautious overall interpretation of results considering objectives, limitations, multiplicity of analyses, results from similar studies, and other relevant evidence | | 18-21 |
| Generalisability | 21 | Discuss the generalisability (external validity) of the study results | | 21-22 |
| Other information | | | | |
| Funding | 22 | Give the source of funding and the role of the funders for the present study and, if applicable, for the original study on which the present article is based | | 24 |

**Give information separately for exposed and unexposed groups.*

**Table B.** Model hyperparameters and quality reports for SVR-symptom mapping analyses. Optimization of hyperparameters was performed using Bayesian optimization with 200 iterations, and SVR-β maps were generated using 5-fold cross-validation.

|  | Variables | Cost/Box Constraint | Sigma/Kernel Scale | Epsilon | Average prediction accuracy | Average reproducibility index |
| --- | --- | --- | --- | --- | --- | --- |
| SVR-LSM | ASRS at FA | 298.80 | 289.48 | 0.06 | 0.35 (0.04) | 0.72 (0.07) |
|  | Naming | 63.64 | 299.86 | 0.01 | 0.22 (0.05) | 0.66 (0.19) |
|  | EF | 99.57 | 121.73 | 1.14 | 0.03 (0.04) | 0.83 (0.09) |
|  | ASRS at DA | 0.81 | 26.21 | 2.99 | -0.24 (0.13) | 0.59 (0.20) |
| SVR-DSM | ASRS at FA | 288.64 | 196.04 | 0.20 | 0.05 (0.17) | 0.86 (0.05) |
|  | Naming | 297.23 | 0.00 | 2.95 | -0.22 (0.09) | 0.83 (0.07) |
|  | EF | 240.13 | 283.47 | 1.95 | -0.01 (0.08) | 0.88 (0.05) |
|  | ASRS at DA | 0.90 | 0.01 | 2.95 | -0.16 (0.14) | 0.64 (0.14) |

ASRS, Aphasia Severity Rating Scale; FA, First Assessment; DA, Discharge Assessment; EF, Executive Functions; SVR-DSM, Support-Vector Regression Disconnectome-Symptom Mapping; SVR-LSM, Support-Vector Regression Lesion-Symptom Mapping.

**Table C.** Results of stepwise linear regression analysis with bootstrap (1,000 replications) to identify covariates for SVR-symptom mapping analyses. For each variable, we presented the percentage of times that the variable was selected as an independent predictor, the sign of the regression coefficient was positive or negative, and the variable was significant in the model. We also presented the final model with t-value and p-value for each variable.

|  | Selection frequency (%) | Sign of coefficient | | Significance (%) | Final model |
| --- | --- | --- | --- | --- | --- |
|  |  | + (%) | – (%) |  |  |
|  |  |  |  |  | **ASRS_DA_ ~ ASRS_FA_ + Lesion_size + Age + SLT_intensity** |
| ASRS at FA | 100.0 | 100.00 | 0.00 | 100.00 | **t = 7.54, p < .001** |
| Lesion size (normalized) | 64.5 | 0.31 | 99.69 | 70.23 | t = -1.72, p = 0.091 |
| Age | 59.9 | 0.00 | 100.00 | 53.76 | t = -1.54, p = 0.13 |
| SLT intensity | 46.6 | 99.57 | 0.43 | 50.64 | t = 1.37, p = 0.18 |
| Delay DA | 23.2 | 15.09 | 84.91 | 38.79 |  |
| Stroke subtype | 19.1 | 21.99 | 78.01 | 37.42 |  |
| Sex | 16.1 | 39.88 | 60.12 | 37.17 |  |
|  |  |  | |  | **ASRS_FA_ ~ Lesion_size + Age + Delay_FA + Sex** |
| Lesion size (normalized) | 100.0 | 0.00 | 100.00 | 100.00 | **t = -5.55, p < .001** |
| Delay FA | 61.8 | 0.32 | 99.68 | 64.89 | t = -1.64, p = 0.11 |
| Age | 60.6 | 0.33 | 99.67 | 64.69 | t = -1.61, p = 0.11 |
| Sex | 56.3 | 0.53 | 99.47 | 65.90 | t = -1.45, p = 0.15 |
| Stroke subtype | 38.4 | 90.89 | 9.11 | 60.68 |  |
|  |  |  | |  | **Naming ~ Lesion_size + Age** |
| Lesion size (normalized) | 100.00 | 0.00 | 100.00 | 100.00 | **t = -6.13, p < .001** |
| Age | 76.5 | 0.13 | 99.87 | 68.63 | **t = -2.03, p = 0.047** |
| Stroke subtype | 38.1 | 95.01 | 4.99 | 46.46 |  |
| Sex | 20.6 | 86.89 | 13.11 | 35.44 |  |
|  |  |  |  |  | **EF ~ Age + Stroke_subtype** |
| Age | 95.5 | 100.00 | 0.00 | 92.98 | **t = 3.21, p = 0.0020** |
| Stroke subtype | 85.5 | 0.12 | 99.88 | 84.56 | **t = -2.95, p = 0.0044** |
| Sex | 37.8 | 3.17 | 96.83 | 50.00 |  |
| Lesion size (normalized) | 25.3 | 93.68 | 6.32 | 37.15 |  |

ASRS, Aphasia Severity Rating Scale; FA, First Assessment; DA, Discharge Assessment; SLT, Speech and Language Therapy.

**Table D.** Sociodemographic and clinical characteristics of the 72 patients with aphasia after a left-hemispheric stroke. Sociodemographic and clinical characteristics, and the intensity of speech and language therapy, were also presented for the 56 patients selected to evaluate aphasia outcome.

|  | All patients (N=72) | Patients for outcome (N=56) |
| --- | --- | --- |
| Age (years) | 64.11 (54.12-70.72) | 64.11 (54.48-70.72) |
| Sex, Male (%) | 47 (65.28) | 38 (67.86) |
| Handedness, RH (%) | 68 (94.44) | 53 (94.64) |
| Stroke subtype, ischemic (%) | 57 (79.17) | 47 (83.93) |
| Lesion size (in voxels) | 21,732 (7,386-48,090) | 24,401 (7,727-50,693) |
| ASRS at FA (0-5) | 2 (1-3) | 2 (1-3) |
| Delay FA (in days post-stroke) | 19.00 (11.75-26.50) |  |
| Naming at FA (0-100%) | 70.00 (18.75-85.00) |  |
| Executive dysfunction at FA (0-3) | 2 (1-2) |  |
| SLT intensity (in days per week) |  | 4.40 (2.23-5.00) |
| Delay DA (in days post-stroke) |  | 85.50 (62.00-123.50) |

*Data is displayed as either the median along with the interquartile range (Q1-Q3) or as a count with percentage (%).*

*RH, Right Handers; ASRS, Aphasia Severity Rating Scale; FA, First Assessment; DA, Discharge Assessment; SLT, Speech and Language Therapy.
Data missing: naming at FA (N=11), SLT intensity (N=2).*
